# A237 STATIN USE IN LIVER TRANSPLANT: INDICATION, USE, AND IMPACT OF STATIN THERAPY ON PATIENT OUTCOMES

**DOI:** 10.1093/jcag/gwac036.237

**Published:** 2023-03-07

**Authors:** M K Smith, C Laidlaw, J G Abraldes, R Bhanji

**Affiliations:** 1 Division of Gastroenterology; 2 Faculty of Medicine and Dentistry; 3 Liver Unit, Division of Gastroenterology, University of Alberta, Edmonton, Canada

## Abstract

**Background:**

Cardiovascular and metabolic diseases are prevalent among patients with chronic liver disease (CLD) and contribute to adverse outcomes and mortality. Prior studies have hypothesized statin therapy improves liver transplant (LT) survival due to anti-inflammatory properties and reduced cardiac risk. Evidence for use of statins in this population is limited as acute liver failure or decompensated cirrhosis is considered a contraindication. Despite evidence supporting statin therapy in CLD their use remains inconsistent.

**Purpose:**

Our study aimed to evaluate the indication and rate of statin use in patients both pre- and post-LT, as well as the impact on post-LT patient survival, graft failure, and post-LT cardiovascular and metabolic disease.

**Method:**

This was a retrospective cohort study of adult LT recipients at the University of Alberta, Edmonton, Canada between 2005 and 2020. Exclusion criteria included pre-transplant acute liver failure, multi-visceral transplant, and re-transplant. Demographic, pharmaceutical and clinical data was collected through review of electronic medical records. Primary endpoints included rate of statin use, post-LT patient survival, and graft failure; secondary endpoints included development of cardiac disease, dyslipidemia, and metabolic syndrome post-LT.

**Result(s):**

We identified 868 patients meeting inclusion criteria; 596 (68.7%) were male, 713 (82.3%) were Caucasian, and median age at transplant was 55. The most common indication for LT was hepatocellular carcinoma in 291 patients (33.5%), followed by Hepatitis C Virus (24.0%), and alcohol liver disease (16.9%). There were no significant demographic differences between those who were and were not on statin therapy. In the pre-LT period only 3% of patients were on statins, despite 7% of patients having dyslipidemia and 24% having diabetes. A total of 261 patients (29.9%) were placed on statins post-LT. The use of statins post-LT was associated with decreased mortality (OR 0.433, 95% CI [0.302-0.622], p<0.001) and decreased graft failure (OR 0.398, 95% CI [0.276-0.574], p<0.001), however it was also associated with increased graft rejection (OR 1.40, 95% CI [1.02-1.93], p=0.039). There were no significant differences in the development of dyslipidemia, metabolic syndrome or cardiac events in post-LT patients regardless of statin use.

**Conclusion(s):**

In our study we identified an association between statin use in the post-LT setting and improved mortality and graft survival. We did not find an association with improved cardiac or metabolic outcomes. The negative association between statin use and post-LT rejection may reflect detection bias as patients with rejection are closely monitored. We also identified a discrepancy between the number of patients with indication for statin use and those who were on statins both pre and post LT, reflecting underutilization consistent with prior literature. Additional studies are required to elucidate the role of statin medications in the transplant hepatologists armamentarium.

**Please acknowledge all funding agencies by checking the applicable boxes below:**

None

**Disclosure of Interest:**

None Declared

